# Change in Soil Particle Size Distribution and Erodibility with Latitude and Vegetation Restoration Chronosequence on the Loess Plateau, China

**DOI:** 10.3390/ijerph17030822

**Published:** 2020-01-28

**Authors:** Jiaying Zhai, Yahui Song, Wulan Entemake, Hongwei Xu, Yang Wu, Qing Qu, Sha Xue

**Affiliations:** 1 State Key Laboratory of Soil Erosion and Dryland Farming on the Loess Plateau, Institute of Soil and Water Conservation, Northwest A&F University, Yangling 712100, China; zhaijiaying6@126.com (J.Z.); songyahh@163.com (Y.S.); WuLan_EnTeMaKe@163.com (W.E.); xuhongwei16@mails.ucas.ac.cn (H.X.); wuyang0521@nwsuaf.edu.cn (Y.W.); ylxnqq@nwafu.edu.cn (Q.Q.); 2 Institute of Soil and Water Conservation, Chinese Academy of Sciences and Ministry of Water Resources, Yangling 712100, China; 3 University of Chinese Academy of Sciences, Beijing 100049, China; 4 College of Forestry, Northwest A&F University, Yangling 712100, China

**Keywords:** vegetation restoration, vegetation zone, particle size distribution, multifractal dimensions, soil erodibility

## Abstract

Analyzing the dynamics of soil particle size distribution (PSD) and erodibility is important for understanding the changes of soil texture and quality after cropland abandonment. This study aimed to determine how restoration age and latitude affect soil erodibility and the multifractal dimensions of PSD during natural recovery. We collected soil samples from grassland, shrubland, and forests with different restoration ages in the steppe zone (SZ), forest-steppe zone (FSZ), and forest zone (FZ). Various analyses were conducted on the samples, including multifractal analysis and erodibility analysis. Our results showed that restoration age had no significant effect on the multifractal dimensions of PSD (capacity dimension (D0), information dimension (D1), information dimension/capacity dimension ratio (D1/D0), correlation dimension (D2)), and soil erodibility. Multifractal dimensions tended to increase, while soil erodibility tended to decrease, with restoration age. Latitude was negatively correlated with fractal dimensions (D0, D2) and positively correlated with K and D1/D0. During vegetation restoration, restoration age, precipitation, and temperature affect the development of vegetation, resulting in differences in soil organic carbon, total nitrogen, soil texture, and soil enzyme activity, and by affecting soil structure to change the soil stability. This study revealed the impact of restoration age and latitude on soil erosion in the Loess Plateau.

## 1. Introduction

Excessive and unregulated land reclamation, intensive cultivation, and the loss of vegetation have led to the degradation of millions of hectares of arable land on the Loess Plateau of China. Severe soil erosion has resulted in most of the topsoil being lost in many locations, leading to the degradation of vegetation and the deterioration of the ecological environment [1]. To restore the eco-environment, the Chinese government launched the state-funded “Grain to Green” project in 1999 [2], through which many cropland areas were converted back to forestland or grassland, or were abandoned to allow natural recovery. The large-scale implementation of returning farmland to forests over several years has brought the soil erosion problem under control in some areas of the Loess Plateau [3].

The soil restoration process improved the soil properties and effectively protected soil from erosion, which greatly affects the particle size distribution (PSD) of soil [4]. PSD is a fundamental physical attribute of soil, affecting the movement of moisture in the soil and soil stability [5]. Thus, it is important to characterize variation in soil PSD to understand and quantify the structure and dynamics of soil [6]. The size distribution of soil microaggregates is also a good indicator of changes to soil structure. PSD is often characterized by multifractal analysis [7,8]. The Rényi (Dq) spectrum is useful for obtaining precise information on PSD [9,10]. Many studies have successfully applied multifractal spectra and associated parameters to characterize changes to PSD in soil during the succession process [8,11]. Wang et al. reported that the rank series of multifractal parameters among land uses strongly contrasts with the degree of soil erosion on the Loess Plateau [12]. Sun et al. also reported that information dimension (D1) and correlation dimension (D2) can be used as indicators to quantify changes in soil erosion [13]. Therefore, multifractal analysis can be used to detect changes to soil properties caused by land-use change.

Many studies have reported that the transformation from sloping farmland to forestland and grassland has helped improve soil conditions [13,14]. Vegetation cover has a positive effect on preventing soil erosion, possibly because it causes direct changes to surface runoff or because it indirectly improves soil infiltration performance and reduces erodibility [15,16]. These positive effects of transformations strongly influence the fraction of fine particles by changing soil conditions [4,17]. Many studies have evaluated how vegetation succession processes affect specific soil properties over time [6,18]. However, changes to precipitation and temperature in different vegetation zones also influence soil properties. Temperature and precipitation affect plant development, microbial activity, and organic carbon storage [19,20]. Previous studies have reported that the number of clay particles in soil also increase with latitude [21]. Thus, it is important to clarify the mechanisms regulating how latitude and restoration age influence soil texture and erodibility.

In the hilly region of the Loess Plateau, the terrain is complex and with diverse vegetation types. Based on bioclimate and erosion capacity, the Loess Plateau can be divided from south to north into forest and forest-steppe water erosion zones, grass prototype water erosion wind erosion zones, and desert steppe and desert wind erosion zones [22]. The landscape characteristics of forest vegetation area, forest grassland vegetation area, and grassland vegetation area are formed successively [23]. In order to determine the impact of restoration age and latitude on soil erodibility and the multifractal dimensions of PSD, we collected soil samples from 210 plots and eight sloping cropland plots spanning three vegetation zones. We analyzed soil particle size distribution, soil microaggregate distribution, soil erodibility, and the multifractal dimensions of PSD. We hypothesized that: (1) with increasing time after cropland abandonment, soil erodibility would decrease, and the multifractal dimensions of PSD would increase; and (2) different latitudes have different mechanisms of action on soil erodibility.

## 2. Materials and Methods 

### 2.1. Study Area

**Figure 1:**
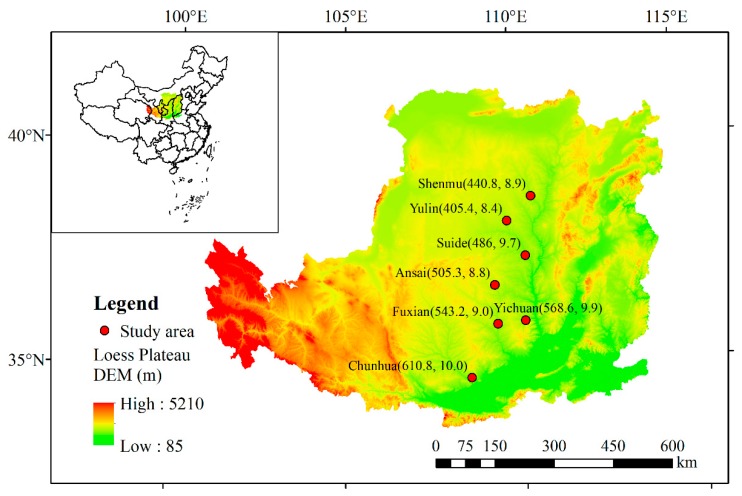
Location of the Loess Plateau in China and the study areas. Mean annual precipitation (mm) and mean annual temperature (°C) are marked beside the study areas.

According to the latitudinal gradient of the Loess Plateau, seven study areas were selected, including the steppe zone (SZ; Shenmu and Yulin), the forest-steppe zone (FSZ; Suide and Ansai), and the forest zone (FZ; Yichuan, Fuxian, and Chunhua; Figure 1). These areas were located along the north-south latitude belt of the Loess Plateau, China (37.01–40.41° N, 109.626–111.78° E). The mean annual precipitation (MAP) and mean annual temperature (MAT) have pronounced gradient characteristics from north to south. MAP ranges from 405.4 to 610.8 mm, while MAT ranges from 8.4 to 10.0 °C. The soil type of FSZ and FZ is mainly loessial soil, while the soil type of SZ is mainly aeolian sandy soil.

### 2.2. Experimental Design

This study used the approach of substituting space for time to investigate the multifractal characteristics and erodibility of PSD in different vegetation zones across the vegetation restoration chronosequence. Seventy sampling sites of different vegetation restoration types were selected in the three vegetation zones as experimental sites (the sampling sites had not been affected by human and herbivore disturbance). The steppe zone (SZ) grassland included 10 sampling sites that had been abandoned for 1, 5, 6, 8, 10, 15, 25, and 30 years. The shrubland and forest of SZ included six sampling sites (abandoned for 10, 25, 30, and 35 years) and seven sampling sites (abandoned for 30, 35, 43, and 45 years), respectively. The forest-steppe zone (FSZ) grassland included six sampling sites that had been abandoned for 7, 17, 20, 25, and 30 years. The shrubland and forest in the FSZ included four sampling sites (abandoned for 10, 25, 30, and 35 years) and 18 sampling sites (abandoned for 8, 12, 14, 24, 25, 26, 28, 30, 33, 38, and 45 years), respectively. The forest zone (FZ) grassland included three sampling sites that has been abandoned for 9, 21, and 30 years. The FZ forest included 16 sampling sites that had been abandoned for 5, 9, 15, 21, 30, 34, 35, 40, and 44 years. Grassland, shrubland, and forest sites in all three vegetation zones were divided into three 10 × 10 m^2^, 10 × 10 m^2^, and 20 × 20 m^2^ plots, respectively. These plots were separated by at least 50 m and were considered to be independent. The selected sites were considered to be representative, typical, and consistent (sites selected in different vegetation zones had similar environmental conditions, such as similar farming practices, topography, slope gradients, slope position, and slope aspects). In addition, two slope cropland plots in SZ, and three slope cropland plots in FSZ and FZ, were selected as reference sites. The basic conditions of the sampling sites are shown in Table S1.

### 2.3. Soil Sampling

In each plot, 10 soil core samples (0–20 cm) were collected by using a soil auger (4 cm diameter), after removing the litter layer, and then the cores were mixed to form a composite sample. After removing roots, stones, and other debris, mixed samples were halved. One half of the sample was naturally air-dried and passed through a 2 mm sieve to determine PSD and microaggregates. An aliquot was then ground and passed through a 0.25 mm sieve to determine soil organic carbon (SOC), total nitrogen (TN), total phosphorus (TP). The other half of the sample was stored at 4 °C to determine soil enzyme activity (soil catalase activity (CAT), soil polyphenol oxidase activity (PPO), soil phosphatase activity (PHO), soil urease activity (URE), and soil saccharase activity (SA)).

### 2.4. Laboratory Analysis

SOC was determined using dichromate oxidation [24]. TN was determined by the Kjeldahl method [25], and TP was determined by molybdenum antimony blue colorimetry [26]. The PSD of soil and microaggregate distribution were measured according to Xiao [3]. When determining soil PSD, samples were pretreated with 6% H_2_O_2_ and 10% HCL, and soaked in distilled water for 24 h. After removing the distilled water, the samples were chemically dispersed with 0.4% Calgon, and were mechanically dispersed in an ultrasonic bath for 5 min. For microaggregates, the samples were soaked in distilled water for 24 h, and placed in an ultrasonic bath for 5 min for mechanical dispersion. PSD and microaggregate distribution were measured by laser diffraction using a Longbench Mastersizer 2000 (Malvern Instruments, Malvern, England). Soil PSD was described in terms of the percentage of clay (<0.001 mm), silt (0.001–0.050 mm), and sand (0.050–1.0 mm). Soil microaggregate distribution was classified into <0.001 mm, 0.001–0.050 mm, and 0.050–1.0 mm grades. Soil enzyme activity was measured using assay techniques modified from Xue et al. [27].

### 2.5. Multifractal Analysis

The generalized fractal dimension (Dq) was applied to express the multifractal characteristics of the soil PSD. The range I = [0.1, 2000] is the measurement interval, which was divided into 64 subintervals Ii = [φ_i_, φ_i+1_], I = 1, 2, …, 64. The sampling intervals were logarithmic arithmetic increments of soil particle size, and log (ϕ_i+1_/ϕ_i_) is constant, the particle size of the first subinterval rage I_1_ = [0.100, 0.117], and I_64_ = [1709.07, 2000]. The transformation φ_j_ = log(ϕ_i+1_/ϕ_1_) for j = 1, 2, . . . 65 creates a new dimensionless interval J = [0, 4.301], partitioned into 64 subintervals of equal length [28]. ε then has a value of J × 2^−k^ for k, ranging from 1 to 6, where ε = 2.15–0.07.

The generalized fractal dimension, Dq, is calculated as [29]:

(1) $$D_{\left(q\right)}=\frac{1}{q-1}\underset{\varepsilon \to 0}{\lim}\frac{\log\left[\sum_{i=1}^{N\left(\varepsilon \right)}\mu_{i}^{q}\left(\varepsilon \right)\right]}{\log\varepsilon }\left(q\neq 1\right)$$

and

(2) $$D_{1}=\underset{\varepsilon \to 0}{\lim}\frac{\sum_{i=1}^{N\left(\varepsilon \right)}\mu_{i}\left(\varepsilon \right)\log\left[\mu_{i}\left(\varepsilon \right)\right]}{\log\varepsilon }\left(q=1\right)$$

For Dq, when q = 0, 1, 2, the corresponding D0, D1, and D2 are known as capacity dimension, information dimension, and correlation dimension, respectively [9]. D0 reflects the distribution range of PSD [30]. D1 is a measure of the heterogeneity of PSD [12]. D1/D0 reflects more singularity information of soil PSD. The closer D1/D0 is to 1, the more concentrated the surface particles are in the dense area. In comparison, the closer D1/D0 is to 0, the more concentrated the particles are in the sparse area. D2 describes the uniformity of the measured values among intervals.

### 2.6. Erodibility (K)

Soil erodibility was measured by the *K* factor in the EPIC model using SOC content and soil PSD [31] and was calculated as:

(3) $$K=\left\{0.2+0.3\exp\left[-0.0256\mathrm{SAN}\left(1-0.01\mathrm{SIL}\right)\right]\right\}\times {\left(\frac{\mathrm{SIL}}{\mathrm{CLA}+\mathrm{SIL}}\right)}^{0.3}\times \left(1.0-\frac{0.25C}{C+\exp\left(3.72-2.95C\right)}\right)\times \left(1.0-\frac{0.75\mathrm{SNI}}{\mathrm{SNI}+\exp\left(-5.51+22.9\mathrm{SNI}\right)}\right)$$

where SAN is the sand content, SIL is the silt content, and CLA is the clay content. SNI = 1-SAN/100, C is the SOC content.

### 2.7. Percentage Contribution

The output from a univariate analysis of variance (ANOVA) was used to statistically assess the influence that each investigated factor had on multifractal dimensions and soil erodibility. The percentage contribution (PC) of each factor was calculated by using the equation:

(4) $$\mathrm{PC}=\frac{{\mathrm{SS}}_{F}-\left(\mathrm{DF}\times V_{\mathrm{Er}}\right)}{{\mathrm{SS}}_{T}}\times 100$$

where SS_T_ is the total sum of squares, SS_F_ is the factorial sum of squares, V_Er_ is the variance of error, and DF is the degrees of freedom. Analysis of Variance output gives the values of DF, SS_T_, SS_F_, and V_Er_ [32].

### 2.8. Statistical Analysis

Linear regression analysis was used to analyze the relationship of multifractal dimensions and erodibility factors with restoration age and latitude. All statistical analyses used IBM SPSS Statistics 22.0 (International Business Machines Corporation, Armonk, USA). *p* < 0.05 indicated that the difference was statistically significant. Figures were drawn using Origin 2018 (OriginLab Corporation, Northampton, USA). Multifractal dimensions were calculated and drawn in R version 3.6.1 (R Core Team, 2019). (R: A language and environment for statistical computing. R Foundation for Statistical Computing, Vienna, Austria. URL https://www.R-project.org). Principal component analysis (PCA) was used to compare the difference of soil particle size distribution in different vegetation zones using R packages “FactoMineR” and “factoextra”, and the multiresponse permutation procedures (MRPP significance) test using the R “vegan” package. Redundancy analysis (RDA) was used to determine the relationships among environmental variables (soil physicochemical and biological factors and environmental factors) and species variables (soil erodibility factor and multifractal dimensions) at different latitudes. Before the RDA, gradient lengths were measured by detrended correspondence analysis (DCA). As the first gradient length was <3 (0.89), a linear method was applied. The red line represents environmental variables and the blue line represents species variables. Acute, obtuse, and right angles between arrows indicate positive, negative, and no correlation, respectively. The RDA was performed using CANOCO 5.0 (Biometris, Plant Research International, Wageningen University and Research Centre, Wageningen, the Netherlands and Petr Šmilauer, Ceske Budejovice, Czech Republic). 

## 3. Results

### 3.1. Changes to the PSD of Soil and Soil Microaggregate Distribution in the Vegetation Zones after Cropland Abandonment

**Figure 2:**
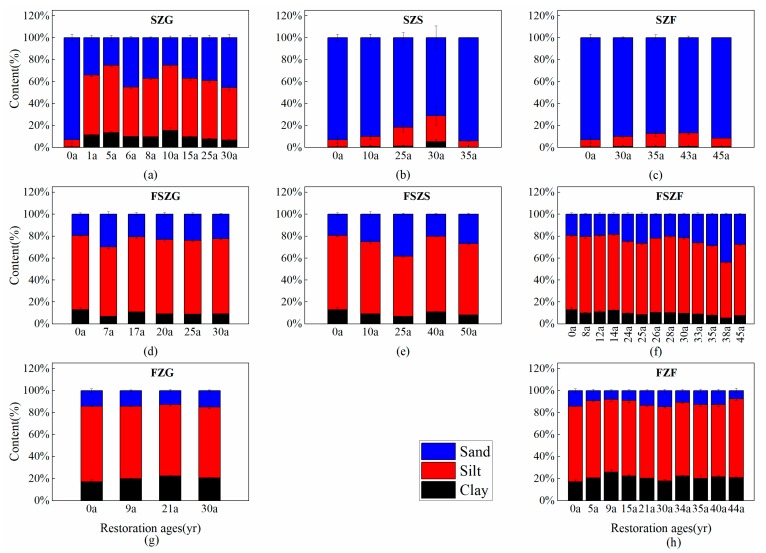
Changes to the PSD of soil in different vegetation zones across the vegetation restoration chronosequence. (**a**–**c**) show changes to particle size distribution (PSD) of soil with restoration age in the steppe zone grassland (SZG), steppe zone shrubland (SZS), and steppe zone forest (SZF), respectively; (**d**–**f**) show changes to PSD with restoration age in the forest-steppe zone grassland (FSZG), forest-steppe zone shrubland (FSZS), and forest-steppe zone forest (FSZF), respectively; (**g**–**h**) show variation in the PSD of grassland (FZG) and forest (FZF) with restoration age in the forest zone, respectively. Note: values are mean ± standard error.

**Figure 3:**
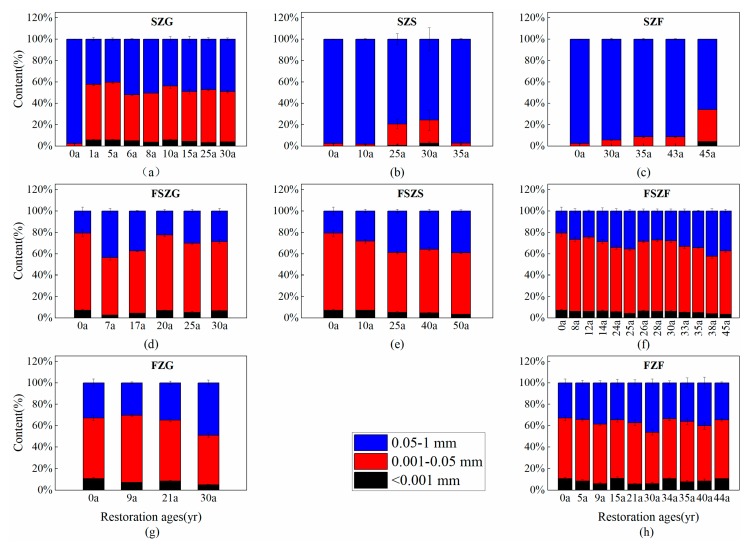
Changes to soil microaggregate content in different vegetation zones across the vegetation restoration chronosequence. (**a**–**c**) show changes to the soil microaggregate content of SZG, SZS, and SZF with restoration age, respectively; (**d**–**f**) show changes to the soil microaggregate content of FSZG, FSZS, and FSZF with restoration age, respectively; (**g**–**h**) show variation in soil microaggregate content of grassland and forests with restoration age in the forest zone, respectively. Note: values are mean ± standard error.

Vegetation restoration had a significant impact on the PSD of soil and the distribution of soil microaggregates following cropland abandonment (Figure 2 and Figure 3). In the forest-steppe zone and forest zone, silt had the highest particle size fraction (49.39–74.05%), followed by sand (3.73–45.58%) and clay (5.06–31.09%). In comparison, sand content was highest in the steppe zone, followed by silt and clay. The distribution of soil microaggregates followed the same trend as soil particle size. Soil microaggregates of 0.001–0.05 mm had the highest fraction (34.0–74.40%), followed by soil microaggregates of 0.05–1 mm (18.88–54.93%). Size classes of <0.001 mm diameter (1.06–17.32%) contained the least material in the forest-steppe zone and forest zone. However, 0.05–1 mm microaggregates had the highest content, followed by 0.001–0.05 mm, and <0.001 mm in the steppe zone. Clay and <0.001 mm microaggregates were ordered: FZ > FSZ > SZ. Sand and <0.001 mm microaggregates were ordered: SZ > FSZ > FZ. The proportion of clay-sized particles increased with vegetation restoration; however, the proportion of sand-sized particles decreased with restoration age. The distribution of soil microaggregates in different vegetation areas and restoration age showed the same trend.

### 3.2. Changes to Multifractal Parameters and Erodibility Factors in the Vegetation Zones Across the Vegetation Restoration Chronosequence

**Figure 4:**
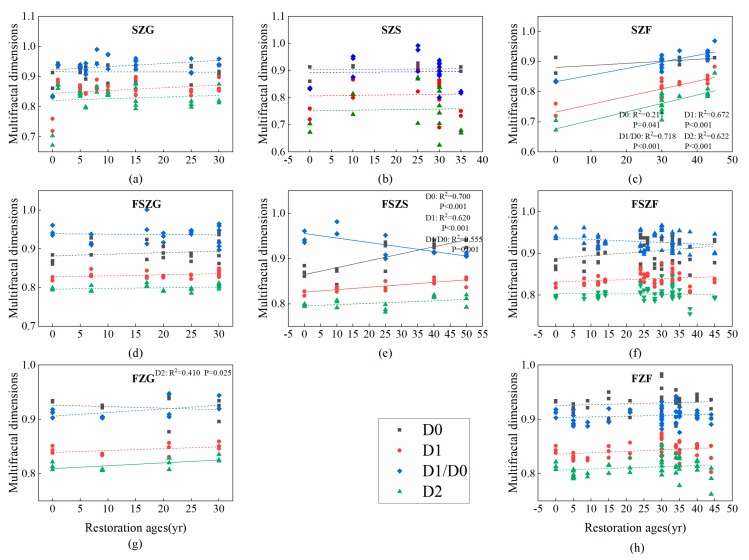
Changes to the soil multifractal parameters of the vegetation zones across the vegetation restoration chronosequence. (**a**–**c**) show changes to the soil multifractal parameters of SZG, SZS, and SZF with restoration age, respectively; (**d**–**f**) show changes to the soil multifractal parameters of FSZG, FSZS, and FSZF with restoration age, respectively; (**g**–**h**) show variation in soil multifractal parameters of grassland and forests with restoration age in the forest zone, respectively. D0: capacity dimension; D1: information dimension; D1/D0: information dimension/capacity dimension ratio; D2: correlation dimension.

**Figure 5:**
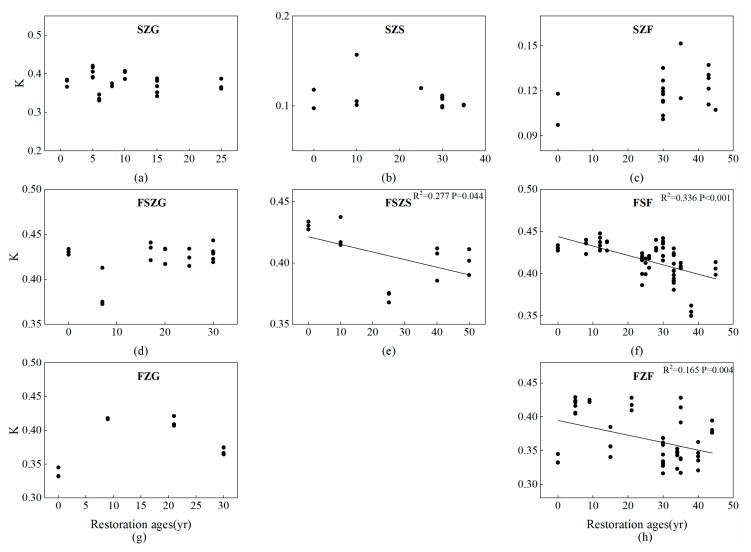
Changes to the erodibility factor of the vegetation zones across the vegetation restoration chronosequence. (**a**–**c**) show changes to the *K* factor of SZG, SZS, and SZF with restoration age, respectively; (**d**–**f**) show changes to the *K* factor of FSZG, FSZS, and FSZF with restoration age, respectively; (**g**–**h**) show changes to the *K* factor of grassland and forests varied restoration age in the forest zone, respectively.

D1 and D2 had an increasing tendency with the succession proceeded (Figure 4). The effect of restoration age on D0 was not significant but increased significantly in SZF and FSZS. D1/D0 increased for all vegetation types in SZ and FZ but decreased for all vegetation types in FSZ. The *K* factor did not change consistently with age; however, significant negative linear relationships occurred between the *K* factor and restoration age for shrubland and forest in FSZ and forest in FZ, respectively (Figure 5). Grassland in all vegetation zones, and shrubland and forest in the SZ did not change significantly after cropland abandonment.

**Figure 6:**
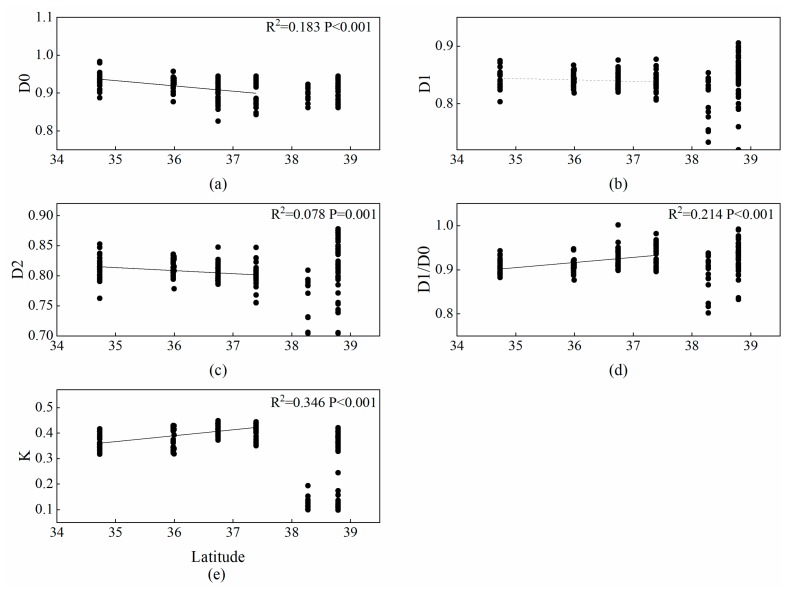
Changes to multifractal dimensions and the erodibility factor with latitude. (**a**–**e**) show the variation of D0, D1, D2, D1/D0, and *K* with latitude respectively.

Correlation analysis showed that latitude significantly affected multifractal dimensions and the erodibility factor. The soil type of FSZ and the FZ in the latitude range form 34.7–37.4 is mainly loessial soil, and the soil type of SZ in the latitude range form 38.2–38.8 is mainly aeolian sandy soil. Because soil texture differs between the high-latitude steppe zone and low-latitude forest-steppe zone and forest zone, we divided latitude into two parts when analyzing multifractal parameters and the erodibility factor. The D0 and D2 of multifractal dimensions in the low latitude zones had a significantly negative correlation with latitude (*p* < 0.001, *p* = 0.001). In comparison, D1/D0 and *K* were positively correlated with latitude (*p* < 0.001, *p* < 0.001). D1 did not significantly change after cropland abandonment (Figure 6). 

### 3.3. Factors Influencing Soil Erodibility and Multifractal Dimensions

**Figure 7:**
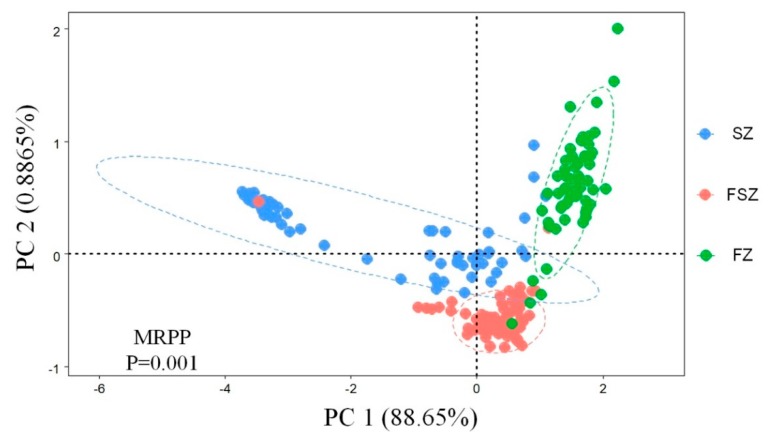
The difference of soil particle size distribution in different vegetation zones.

**Figure 8:**
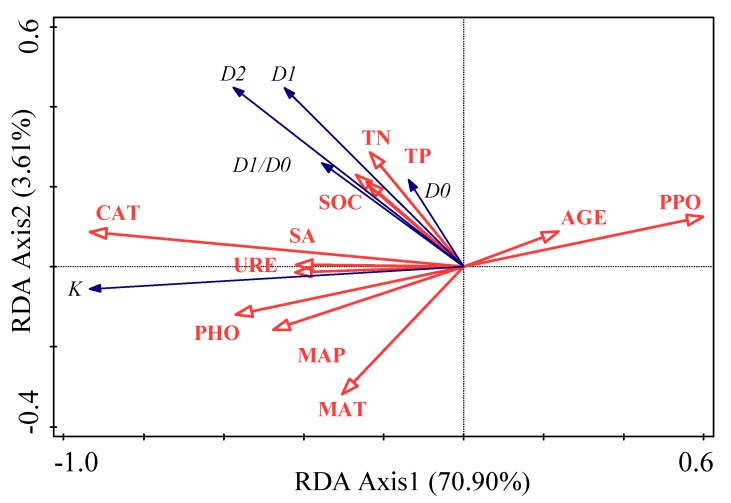
Ordination plot of the redundancy analysis (RDA) for soil erodibility and multifractal dimensions with soil properties as constraining variables. D0 is the capacity dimension; D1 is the information dimension; D2 is the correlation dimension; D1/D0 is the information dimension/capacity dimension ratio; *K* is the erodibility factor; SOC is soil organic carbon; TN is total nitrogen; TP is total phosphorus; AGE is restoration age; MAP is mean annual precipitation; MAT is mean annual precipitation; CAT is soil catalase activity; PPO is soil polyphenol oxidase activity; PHO is soil phosphatase activity; URE is soil urease activity; SA is soil saccharase activity.

PCA was used to compare the difference of soil particle size distribution in different vegetation zones (Figure 7), and there were significant differences in soil particle size distribution in different vegetation zones. Univariate ANOVA determined the statistical significance of each factor (latitude and restoration age) on the multifractal dimensions of PSD and soil erodibility (Table 1). According to Equation (4) and the results of the Univariate ANOVA, the percentage contribution (PC) of latitude and restoration age to the multifractal dimensions of PSD and soil erodibility was determined. The factors were ranked in the order of their PC as follows: (1) latitude (58.5%, 16.3%, 18.5%, 21.6%, and 17.1%); (2) restoration age (6.2%, 3.55%, 7.0%, 4.8%, and 3.4%). The relationships among the soil erodibility factor, multifractal dimensions, soil physicochemical properties index, soil biological factors, and environmental factors were explored by RDA (Figure 8). Environmental factors and soil physicochemical and biological properties explained 76.2% of variation in soil erodibility and multifractal dimensions. The first two axes explained 70.9% and 3.6% of variation, respectively. CAT explained 62.0% of variation while SOC, MAP, TN, URE, and TP explained 9.7%, 1.5%, 0.6%, 0.7%, and 0.5% of variation, respectively. The RDA model showed that MAP, MAT, SOC, TN, TP, URE, SA, PHO, and CAT had positive correlations with D0, D1, D1/D0, *K*, and D2. In comparison, restoration age (AGE) and PPO were negatively correlated with D0, D1, D1/D0, *K*, and D2. 

## 4. Discussion

### 4.1. Response of Soil Erodibility and Multifractal Dimensions to Restoration Age and Driving Factors

PSD affects soil fertility and is an important component of soil quality. The current study showed that the multifractal dimensions D1, D2, D1/D0 in SZ and FSZ, and D0 in FSZ, SZS, SZF, and FZ, slightly increased. In comparison, the erodibility factor decreased with increasing restoration age in certain vegetation zones. These findings reflect those of Sun et al. [4]. The decrease in D1/D0 in FSZ with number of years of recovery meant that PSD density declined due to an increase in clay and sand content, which was not only concentrated in areas of silt. The increase in D0, D1, and D2 meant that PSD covered a wider range, with higher heterogeneity and uniformity of measurement intervals, which was attributed to the increase in fine particle content. With the cessation of agricultural practices, clay content and microaggregate content of <0.001 mm increased with the number of recovery years [33]. The increase of fine soil particles might be attributed to the overall effect of improved soil quality and reduced soil erosion reduction after vegetation restoration [13,34]. In general, plant coverage, root system development, the number of root systems, and plant productivity gradually increase with recovery time [35]. High vegetation coverage after farmland abandonment strongly affects soil erosion. The vegetation layer affects erosion by intercepting rainfall and reducing the capacity for wind and water to be transported to the sediment, thus retaining fine particles [12,36,37]. SOC increased over time, which was mainly due to the large amount of soil nutrients released by residues [38,39]. SOC binds to soil particles to form a “spring” that prevents mechanical deformation within and between soil aggregates, in addition to promoting the structuring of soil and higher infiltration capacity. In turn, this phenomenon increases the amount of microaggregates and fine particles [40,41,42,43]. The increase of soil organic matter and decrease of soil erosion generally improves soil structure and increases the amount of microaggregates and fine particles. These phenomena might result in the wider distribution of PSD in the soil, with higher irregularity and heterogeneity. Returning farmland to a more natural state could facilitate the accumulation of organic matter and improve soil structure, thus enhancing soil stability.

### 4.2. Response of Soil Erodibility and Multifractal Dimensions to Latitude and Driving Factors 

Our results showed that there were significant differences in particle size distribution in SZ, FSZ and FZ, and the comparison of contribution percentages indicated that the zonal change was greater than the change brought by vegetation restoration. There are typical regional differences in soil particle distribution because soil particle size distribution on the Loess Plateau is mainly controlled by the eolian deposit of loess parent material and the slow soil-forming process under cold and dry conditions [44,45,46]. Although the contribution of vegetation restoration is small, it has a significant impact on the multifractal dimensions of PSD and soil erodibility. Soil erodibility and D1/D0 significantly increased with increasing latitude, whereas D0 and D2 significantly decreased with increasing latitude, in the forest-steppe zone and the forest zone. The decrease of D0 and D2 with increasing latitude resulted in the particle distribution range becoming narrow and nonuniform. In comparison, the increase of D1/D0 with latitude resulted in an increased concentration of soil in dense areas. Secondary recovery is a complicated process. During vegetation restoration, precipitation, temperature, and the vegetation fraction could alter the texture and erodibility of soil. Changes to precipitation affect soil wind erosion through soil moisture content, leading to differences in soil texture and other soil properties [47,48,49]. Water is a limiting factor for the development of vegetation on the Loess Plateau. Carbon and nitrogen inputs are limited in water-limited areas [50], while litter decomposition and microbial activity in soil water are strongly affected by soil water [51]. Changes to precipitation and temperature gradients led to a wider, and more uniform, distribution of soil PSD with increasing latitude. SOC appears to be an important soil property regulating soil erodibility [52]. As a binder, soil organic matter promotes soil structure and increases its permeability. As a result, the content, range, and density of fine particles increases, which reduces soil erodibility [43]. RDA showed that soil enzyme activity was also the dominant factor driving changes to soil erodibility and multifractal dimensions. The soil surface water and temperature conditions in different vegetation zones vary greatly, and the types and quantities of soil microorganisms are different due to the total amount of litters, resulting in great differences in soil enzyme activities in different vegetation zones [53,54]. Increased soil enzyme activity improves soil fertility and soil microbial biomass to some extent, thus improving soil structure and accelerating the succession of converted vegetation positively [55]. Furthermore, soil stability was improved.

## 5. Conclusions

In the present study, the effect of restoration age on the multifractal dimension and soil erodibility was not significant. Multifractal dimensions tended to increase, while and soil erodibility tended to decline with restoration age. Latitude was negatively correlated with fractal dimensions (D0, D2) and positively correlated with *K* and D1/D0. SOC, TN, TP, soil enzyme activity, precipitation, and temperature had a combined effect on soil erodibility and multifractal dimensions. This study revealed the impact of restoration age and latitude on soil erosion in the Loess Plateau.

## Figures and Tables

**Table 1:** Significance and percentage contribution of the factors’ effects on the multifractal dimensions of soil particle size distribution and soil erodibility based on univariate analysis of variance. D0: capacity dimension; D1: information dimension; D1/D0: information dimension/capacity dimension ratio; D2: correlation dimension.

Variable	Source	Type III Sum of Squares	Degree of Freedom	Mean Sum of Square	F value	*p*	Percentage Contribution/%
*K*	Latitude	1.386	6	0.231	63.675	<0.01	58.5
	Restoration age	0.162	5	0.032	8.915	<0.01	6.2
	Error	0.722	199				32.7
	Total	28.209	211				
D0	Latitude	0.029	6	0.005	8.061	<0.01	16.3
	Restoration age	0.008	5	0.002	2.841	0.017	3.5
	Error	0.118	199				80.9
	Total	175.658	211				
D1	Latitude	0.033	6	0.005	9.314	<0.01	18.5
	Restoration age	0.014	5	0.003	4.764	<0.01	7.0
	Error	0.116	199				77.8
	Total	148.718	211				
D2	Latitude	0.063	6	0.011	11.136	<0.01	21.6
	Restoration age	0.018	5	0.004	3.715	0.003	4.8
	Error	0.188	199				74.4
	Total	136.451	211				
D1/D0	Latitude	0.033	6	0.006	8.638	<0.01	17.1
	Restoration age	0.009	5	0.002	2.833	0.017	3.4
	Error	0.127	199				78.3
	Total	178.913	211				

## Supplementary Materials

The following are available online at https://www.mdpi.com/1660-4601/17/3/822/s1, Table S1: The details of sampling sites.

## References

[1] Zhou Z., Shangguan Z., Zhao D. (2006). Modeling vegetation coverage and soil erosion in the Loess Plateau Area of China. Ecol. Model..

[2] Zhang C., Xue S., Liu G., Song Z. (2011). A comparison of soil qualities of different revegetation types in the Loess Plateau, China. Plant Soil.

[3] Xiao L., Xue S., Liu G., Zhang C. (2014). Fractal features of soil profiles under different land use patterns on the Loess Plateau, China. J. Arid Land.

[4] Sun C., Liu G., Sha X. (2016). Natural succession of grassland on the Loess Plateau of China affects multifractal characteristics of soil particle-size distribution and soil nutrients. Ecol. Res..

[5] Lv Y., Guan X., Ruan B., Wang Y. (2014). Multifractal Characteristics of Soil Particle Size Distribution under Sewage Irrigation in Different Irrigation Years. Appl. Mechan. Mater..

[6] Yu J., Lv X., Bin M., Wu H., Du S., Zhou M., Yang Y., Han G. (2015). Fractal features of soil particle size distribution in newly formed wetlands in the Yellow River Delta. Sci. Rep..

[7] Grout H., Tarquis A.M., Wiesner M.R. (1998). Multifractal Analysis of Particle Size Distributions in Soil. Environ. Sci. Technol..

[8] Posadas A.N.D., Giménez D., Bittelli M., Vaz C.M.P., Flury M. (2001). Multifractal Characterization of Soil Particle-Size Distributions. Soil Sci. Soc. Am. J..

[9] Montero E. (2005). Renyi dimensions analysis of soil particle-size distributions. Ecol. Model..

[10] Paz-Ferreiro J., Vázquez E.V., Miranda J.G.V. (2010). Assessing soil particle-size distribution on experimental plots with similar texture under different management systems using multifractal parameters. Geoderma.

[11] Sun C., Liu G., Sha X. (2016). Response of soil multifractal characteristics and erodibility to 15-year fertilization on cropland in the Loess Plateau, China. Arch. Agro. Soil Sci..

[12] Wang D., Fu B., Zhao W., Hu H., Wang Y. (2008). Multifractal characteristics of soil particle size distribution under different land-use types on the Loess Plateau, China. Catena.

[13] Sun C., Liu G., Xue S. (2016). Land-Use Conversion Changes the Multifractal Features of Particle-Size Distribution on the Loess Plateau of China. Int. J. Environ. Res. Public Health.

[14] Sun W., Shao Q., Liu J., Zhai J. (2014). Assessing the effects of land use and topography on soil erosion on the Loess Plateau in China. Catena.

[15] Yang M. (2014). Study on Soil Erodibility in Arid Valley Core Area of Upper Minjiang River. Master’s Thesis.

[16] Liu Q., Li Z., Li P., Han J. (2012). On Soil Erodibility of Purple soil in Small Watersheds in Sichuan Basin. J. Mianyang Teach. Coll..

[17] Wang Y., Zhou G., Jia B. (2008). Modeling SOC and NPP responses of meadow steppe to different grazing intensities in Northeast China. Ecol. Model..

[18] Xia L., Zhang G., Heathman G.C., Wang Y., Huang C.H. (2009). Fractal features of soil particle-size distribution as affected by plant communities in the forested region of Mountain Yimeng, China. Geoderma.

[19] Zhu Z., Huang Y., Xu F., Xing W., Zheng S., Bai Y. (2017). Effects of precipitation intensity and temporal pattern on soil nitrogen mineralization in a typical steppe of Nei Mongol grassland. Chin. J. Plant Ecol..

[20] Zhou T., Shi P., Wang S. (2003). Impacts of Climate Change and Human Activities on Soil Carbon Storage in China. Acta Geogr. Sin..

[21] Liu C. (2017). Temporal and Spatial Variation of Soil Organic Carbon and Its Influening Factors in Western Songnen Plain. Master’s Thesis.

[22] Tang K., Hou Q., Wang B., Zhang P. (1993). The Environment Background and Administration Way of Wind-water Erosion Crisscross Region and Shenmu Experimental Area on the Loess Plateau. Soil Water Conser. Res..

[23] Loess Plateau Data Center, National Earth System Science Data Sharing Infrastructure, National Science & Technology Infrastructure of China. http://loess.geodata.cn.

[24] Nelson D.W., Sommers L.E., Sparks D.L., Page A.L., Helmke P.A., Loeppert R.H., Soltanpour P.N., Tabatabai M.A., Johnston C.T., Sumner M.E. (1996). Total carbon, organic carbon, and organic matter. Method. Soil Anal..

[25] Bremner J.M., Sparks D.L., Page A.L., Helmke P.A., Loeppert R.H., Soltanpour P.N., Tabatabai M.A., Johnston C.T., Sumner M.E. (1996). Nitrogen—Total. Method. Soil Anal. Chem. Method. Part.

[26] Murphy J., Riley J.P. (1962). A modified single solution method for the determination of phosphate in natural waters. Anal. Chim. Acta.

[27] Xue S., Yang X., Liu G., Gai L., Zhang C., Ritsema C.J., Geissen V. (2017). Effects of elevated CO_2_ and drought on the microbial biomass and enzymatic activities in the rhizospheres of two grass species in Chinese loess soil. Geoderma.

[28] Miranda J.G.V., Montero E., Alves M.C., González A.P., Vázquez E.V. (2006). Multifractal characterization of saprolite particle-size distributions after topsoil removal. Geoderma.

[29] Hentschel H.G.E., Procaccia I. (1983). The infinite number of generalized dimensions of fractals and strange attractors. Physica D Nonlinear Phenomena.

[30] Wang D., Fu B., Lu K., Xiao L., Zhang Y., Feng X. (2010). Multifractal analysis of land use pattern in space and time: A case study in the Loess Plateau of China. Ecol. Complex..

[31] Williams J.R., Jones C.A., Dyke P.T. (1984). A Modeling Approach to Determining the Relationship Between Erosion and Soil Productivity. Trans. Asae.

[32] Zhang F., Wang Z., Yang M. (2015). Assessing the applicability of the Taguchi design method to an interrill erosion study. J. Hydrol..

[33] Wang B., Liu G., Xue S., Zhu B. (2011). Changes in soil physico-chemical and microbiological properties during natural succession on abandoned farmland in the Loess Plateau. Environ. Earth Sci..

[34] Lyu X., Yu J., Zhou M., Ma B., Wang G., Zhan C., Han G., Guan B., Wu H., Li Y. (2015). Changes of soil particle size distribution in tidal flats in the Yellow River Delta. PLoS ONE.

[35] Xue S., Liu G., Pan Y., Dai Q., Zhang C., Na Y. (2009). Evolution of Soil Labile Organic Matter and Carbon Management Index in the Artificial Robinia of Loess Hilly Area. Scientia Agric. Sin..

[36] Trimble S.W., Crosson P. (2000). LAND USE: U.S. Soil Erosion Rates-Myth and Reality. Science.

[37] Meng Z., Dang X., Gao Y., Ren X., Ding Y., Wang M. (2018). Interactive effects of wind speed, vegetation coverage and soil moisture in controlling wind erosion in a temperate desert steppe, Inner Mongolia of China. J. Arid Land.

[38] Guo C., Dannenmann M., Gasche R., Zeller B., Papen H., Polle A., Rennenberg H., Simon J. (2013). Preferential use of root litter compared to leaf litter by beech seedlings and soil microorganisms. Plant Soil.

[39] Xu H., Qu Q., Li P., Guo Z., Wulan E., Xue S. (2019). Stocks and Stoichiometry of Soil Organic Carbon, Total Nitrogen, and Total Phosphorus after Vegetation Restoration in the Loess Hilly Region, China. Forests.

[40] An S., Zheng F., Zhang F., Pelt S.V., Hamer U., Makeschin F. (2008). Soil quality degradation processes along a deforestation chronosequence in the Ziwuling area, China. Catena.

[41] Zhuang J., Mccarthy J.F., Perfect E., Mayer L.M., Jastrow J.D. (2008). Soil Water Hysteresis in Water-Stable Microaggregates as Affected by Organic Matter. Soil Sci. Soc. Am. J..

[42] An S., Huang Y., Zheng F., Yang J. (2008). Aggregate Characteristics During Natural Revegetation on the Loess Plateau. Pedosphere.

[43] Zhu B., Li Z., Li P., Liu G., Xue S. (2010). Soil erodibility, microbial biomass, and physical–chemical property changes during long-term natural vegetation restoration: A case study in the Loess Plateau, China. Ecol. Res..

[44] Sun J., Zhao J. (1991). Quanternary of Loess Plateau in China.

[45] Yang W., Tian J. (2004). Fractal characteristics of soil particle composition in typical soil profile of loess plateau. Acta Pedol. Sin..

[46] Ding Z., Yu Z., Yang S., Sun J., Xiong S., Liu T. (2001). Coeval changes in grain size and sedimentation rate of eolian loess, the Chinese Loess Plateau. Geophys. Res. Lett..

[47] Wei W., Chen L., Fu B., Chen J. (2010). Water erosion response to rainfall and land use in different drought-level years in a loess hilly area of China. Catena.

[48] Ma L., Yuan S., Guo C., Wang R. (2014). Carbon and nitrogen dynamics of native Leymus chinensis grasslands along a 1000 km longitudinal precipitation gradient in northeastern China. Biogeosci. Discuss..

[49] Wu F., Cao Y., Lu G., Yang J., Zhang T. (2016). Impact Factors of Soil Wind Erosion and Estimation of Soil Loss in Zhundong, Xinjiang. J. Soil Water Conser..

[50] Zhang J., Dong Y. (2010). Factors affecting species diversity of plant communities and the restoration process in the loess area of China. Ecol. Eng..

[51] Bell C., Mcintyre N., Cox S., Tissue D., Zak J. (2008). Soil Microbial Responses to Temporal Variations of Moisture and Temperature in a Chihuahuan Desert Grassland. Microb. Ecol..

[52] Li S., Li Y., Duan S., Zhou X. (2009). Study on Changes of Soil Physiochemical Property Under Converting Cropland to Forest in Datong of Qinghai Province. Res. Soil Water Conser..

[53] Deng L., Peng C., Huang C., Wang K., Liu Q., Liu Y., Hai X., Shangguan Z. (2019). Drivers of soil microbial metabolic limitation changes along a vegetation restoration gradient on the Loess Plateau, China. Geoderma.

[54] Li X., Ma R., An S., Zeng Q., Li Y. (2015). Characteristics of organic carbon and enzyme activities in soil aggregates under different vegetation zones on the loess plateau. Chin. J. Appl. Ecol..

[55] Zhang Y., Du H., Zhang Z., Feng C. (2014). Evolution Characteristics of Soil Biological Property in Loess Hilly Region under Natural Vegetation Restoration. Res. Soil Water Conser..

