# Supplementary material for: Change in Soil Particle Size Distribution and Erodibility with Latitude and Vegetation Restoration Chronosequence on the Loess Plateau, China
Source: Int J Environ Res Public Health. 2020 Jan 28;17(3):822. doi: 10.3390/ijerph17030822 (PMC7037365; doi:10.3390/ijerph17030822)
Supplement: Supplementary file 1 [file ijerph-17-00822-s001.pdf]

Table S1. The details of sampling sites.

Vegetation Zones	Vegetational Types	Sampling Sites	Restoration Ages	Altitude	Slope (°)	Dominant Community Species	Soil Types
SZ	Slope cropland	S1	0	1203	18	*Setaria italica*	Aeolian sandy soil
	Grassland	S2	1	1202	20	*Artemisia scoparia, xeris denticulata*	Aeolian sandy soil
		S3	5	1214	18	*Stipa bungeana, Lespedeza bicolor, Artemisia scoparia*	Aeolian sandy soil
		S4	6	1239	15	*Stipa bungeana, Lespedeza bicolor, Artemisia apillaris*	Aeolian sandy soil
		S5	8	1139	15	*Stipa bungeana, Lespedeza bicolor, xeris denticulata*	Aeolian sandy soil
		S6	10	1214	20	*Artemisia capillaris, Stipa bungeana, Medicago*	Aeolian sandy soil
		S7	15	1227	18	*Stipa bungeana, Lespedeza bicolor, Artemisia apillaris*	Aeolian sandy soil
		S8	25	1235	17	*Bothriochloa ischaemum, Stipa bungeana*	Aeolian sandy soil
		S9	30	1135	16	*Bothriochloa ischaemum, Stipa bungeana, Lespedeza bicolor Turcz*	Aeolian sandy soil
	Shrubland	S10	10	1186	10	*Caragana korshinskii*	Aeolian sandy soil
		S11	25	1198	10	*Caragana korshinskii*	Aeolian sandy soil
		S12	30	1175	10	*Caragana korshinskii*	Aeolian sandy soil
		S13	35	1098	8	*Caragana korshinskii*	Aeolian sandy soil
	Forest	S14	30	1170	19	*Populus simonii*	Aeolian sandy soil
		S15	35	1097	3	*Sabina vulgaris*	Aeolian sandy soil
		S16	43	1100	4	*Pinus sylvestris, Pinus tabuliformis*	Aeolian sandy soil
		S17	45	1214	5	*Robinia pseudoacacia*	Aeolian sandy soil
FSZ	Slope cropland	S18	0	1045	23.5	*Ricinus communis*	Loessial soil
	Grassland	S19	7	1303	32	*Stipa bungeana, Potentilla bifurca, Lespedeza bicolor*	Loessial soil
		S20	17	1303	31	*Stipa bungeana, Lespedeza bicolor, Potentilla bifurca*	Loessial soil
		S21	20	1136	31	*Artemisia leucophylla, Artemisia sacrorum, Lespedeza bicolor, Stipa bungeana*	Loessial soil
		S22	25	1221	27	*Artemisia sacrorum, Stipa bungeana, Lespedeza bicolor*	Loessial soil
		S23	30	1108	32	*Artemisia sacrorum, Stipa bungeana, Lespedeza bicolor*	Loessial soil
	Shrubland	S24	10	913	25	*Caragana korshinskii*	Loessial soil
		S25	25	1144	22	*Caragana korshinskii*	Loessial soil
		S26	40	1221	27	*Caragana korshinskii*	Loessial soil
		S27	50	1090	24	*Caragana korshinskii*	Loessial soil

	Forest	S28	8	1251	29	*Robinia pseudoacacia*	Loessial soil
		S29	12	1233	31	*Robinia pseudoacacia*	Loessial soil
		S30	14	1254	32	*Robinia pseudoacacia*	Loessial soil
		S31	24	1109	26	*Robinia pseudoacacia*	Loessial soil
		S32	25	1260	31	*Robinia pseudoacacia*	Loessial soil
		S33	26	1170	24	*Robinia pseudoacacia*	Loessial soil
		S34	28	1258	27	*Robinia pseudoacacia*	Loessial soil
		S35	30	937	51	*Robinia pseudoacacia*	Loessial soil
		S36	33	1191	27	*Robinia pseudoacacia*	Loessial soil
		S37	35	966	39	*Robinia pseudoacacia*	Loessial soil
		S38	38	1136	31	*Robinia pseudoacacia*	Loessial soil
		S39	45	1198	35	*Robinia pseudoacacia*	Loessial soil
FZ	Slope cropland	S40	0	1040	12	*Setaria italica*	Loessial soil
	Grassland	S41	9	1139	26.5	*Stipa bungeana, Lespedeza bicolor, Artemisia capillaris*	Loessial soil
		S42	21	1034	24	*Artemisia sacrorum, Stipa bungeana, Cleistogenes squarrosajiaohao*	Loessial soil
		S43	30	1048	24	*Artemisia sacrorum, Stipa bungeana, Cleistogenes squarrosa, Artemisia leucophylla*	Loessial soil
	Forest	S44	5	1091	5	*Robinia pseudoacacia*	Loessial soil
		S45	9	1139	50	*Robinia pseudoacacia*	Loessial soil
		S46	15	1023	4	*Robinia pseudoacacia*	Loessial soil
		S47	21	1031	30	*Robinia pseudoacacia*	Loessial soil
		S48	30	1042	15	*Robinia pseudoacacia, Betula paltyphylla, Quercus wutaishanica, Pinus tabuliformis*	Loessial soil
		S49	34	1044	14	*Robinia pseudoacacia, Pinus tabuliformis*	Loessial soil
		S50	35	1038	33	*Robinia pseudoacacia, Platycladus orientalis*	Loessial soil
		S51	40	1124	26	*Robinia pseudoacacia, Pinus tabuliformis*	Loessial soil
		S52	44	1249	11	*Robinia pseudoacacia*	Loessial soil

Note: SZ is steppe zone; FSZ is forest-steppe zone; SZ is forest zone.
